# Genomic and phenotypic insight into antimicrobial resistance of *Pseudomonas fluorescens* from King George Island, Antarctica

**DOI:** 10.3389/fmicb.2025.1535420

**Published:** 2025-03-03

**Authors:** Myllena Pereira Silverio, Júnia Schultz, Mariana T. D. Parise, Doglas Parise, Marcus Vinicius Canário Viana, Wylerson Nogueira, Rommel Thiago Jucá Ramos, Aristoteles Góes-Neto, Vasco Ariston De Carvalho Azevedo, Bertram Brenig, Raquel Regina Bonelli, Alexandre Soares Rosado

**Affiliations:** ^1^Laboratory of Molecular Microbial Ecology, Institute of Microbiology, Federal University of Rio de Janeiro, Rio de Janeiro, Brazil; ^2^Laboratory of Investigation in Medical Microbiology, Institute of Microbiology, Federal University of Rio de Janeiro, Rio de Janeiro, Brazil; ^3^Biological and Environmental Sciences and Engineering Division (BESE), King Abdullah University of Science and Technology (KAUST), Thuwal, Saudi Arabia; ^4^Institute of Biological Sciences, Federal University of Minas Gerais, Belo Horizonte, Brazil; ^5^Institute of Biological Sciences, Federal University of Pará, Belém, Brazil; ^6^Department of Molecular Biology of Livestock, Institute of Veterinary Medicine, Georg August University, Göttingen, Germany; ^7^Bioscience Program, BESE Division, King Abdullah University of Science and Technology (KAUST), Thuwal, Saudi Arabia

**Keywords:** resistomes, psychrotolerant bacteria, Proteobacteria, Pseudomonadota, antibiotics, acquired resistance, intrinsic resistance, efflux pumps

## Abstract

The genus *Pseudomonas* includes metabolically versatile microorganisms occupying diverse niches, from environmental habitats to plant pathogens, and has clinically significant strains. For this reason, *Pseudomonas* spp. might act as a reservoir of antimicrobial resistance genes, which have been detected even in isolated environments. The aim of this study was to report the antimicrobial susceptibility profile of 25 *Pseudomonas fluorescens* isolates from soil samples collected on King George Island (Antarctic Peninsula), and to select non-clonal isolates with unusual phenotypes for whole genome sequencing (WGS). Six classes of antimicrobials were assessed with disk diffusion and colistin with minimum inhibitory concentration (MIC) by broth microdilution. In order to confirm the discrepant phenotypes, MIC by agar dilution was performed for the beta-lactams aztreonam, ceftazidime, cefepime and the aminoglycoside neomycin. The genus *Pseudomonas* was confirmed by matrix-assisted laser desorption/ionization – time of flight (MALDI-TOF) and the clonal relationships were examined using repetitive extragenic palindromic polymerase chain reaction (BOX-PCR), from which 14 strains were selected for WGS. Antimicrobial susceptibility testing revealed that all strains were susceptible to neomycin and exhibited varying degrees of intermediate or full resistance to aztreonam and colistin. Additionally, 11 strains demonstrated intermediate resistance to ceftazidime, and six were resistant to cefepime. The genomic analysis identified various efflux pumps, predominantly from the ABC transporter and resistance-nodulation-division families. Resistance genes were detected against eight classes of antimicrobials, listed by prevalence: beta-lactams, tetracyclines, polymyxins, aminoglycosides, fosmidomycin, fosfomycin, quinolones, and chloramphenicol. Genes associated with heavy-metal resistance, prophages, and adaptations to extreme environments were also investigated. One notable isolate exhibited not only the highest number of pathogenicity and resistance islands, but also presented a carbapenemase-encoding gene (*bla*_PFM-2_) in its genome. Overall, one plasmid was identified in a distinct isolate, which did not exhibit antimicrobial resistance determinants. The genotypic and phenotypic findings are consistent, suggesting that efflux pumps play a critical role in antimicrobial extrusion. This study offers valuable insight into the evolution of antimicrobial resistance in *P. fluorescens*, particularly in extreme environments, such as Antarctica. By exploring the antimicrobial resistance mechanisms in *P. fluorescens*, the study sheds light on how isolated ecosystems drive the natural evolution of resistance genes.

## 1 Introduction

Pseudomonads are ubiquitous and adaptable microorganisms, primarily due to their metabolic versatility and genome plasticity (Craig et al., 2021; Rumbaugh, 2014). The genus is known for its ability to survive cold stress and desiccation (Craig et al., 2021), common environmental conditions in extreme habitats, such as Antarctica. To survive harsh conditions and competition, Pseudomonas has developed an effective response to abiotic stress, including resistance to antimicrobials (Marcoleta et al., 2022; Allen et al., 2009). Therefore, intrinsic resistance in the genus *Pseudomonas* includes altering membrane permeability and overexpressing efflux pumps or chromosomal resistance genes, such as the beta-lactamase gene *bla*AmpC (Silverio et al., 2022; Lupo et al., 2018; Chevalier et al., 2017; Olivares Pacheco et al., 2017; Lima et al., 2015).

The aim of this study was to investigate intrinsic resistance mechanisms in isolates belonging to the *Pseudomonas fluorescens* complex. The isolates were originally from four remote ecosystems in King George Island, Antarctic Peninsula. Antarctica is considered one of the last pristine environments, exhibiting extreme weather conditions, well-preserved ecosystems, and geographical isolation (Cowan et al., 2011). On the other hand, *P. fluorescens* is an opportunistic pathogen that mainly affects immunocompromised patients. These bacteria behave as reservoirs of antimicrobial resistance genes (ARGs), which makes the treatment challenging (Koh et al., 2004; Rolston et al., 2005; Sader and Jones, 2005; Faccone et al., 2014; Montana et al., 2018). Hitherto, few research papers have focused on the antimicrobial susceptibility profile of Antarctic bacteria using whole genome sequencing (WGS). For example, previous works include the beta-lactam-resistant bacteria *Acinetobacter radioresistens* A154 from Fildes Peninsula (Opazo-Capurro et al., 2019) and two methicillin-resistant *Staphylococci* strains from James Ross Island (Pantucek et al., 2018). In this work, 25 *P. fluorescens* isolates were evaluated with phenotypic antimicrobial susceptibility tests, and 14 were selected for WGS.

## 2 Materials and methods

### 2.1 Soil sampling and bacterial isolation

**Figure 1:**
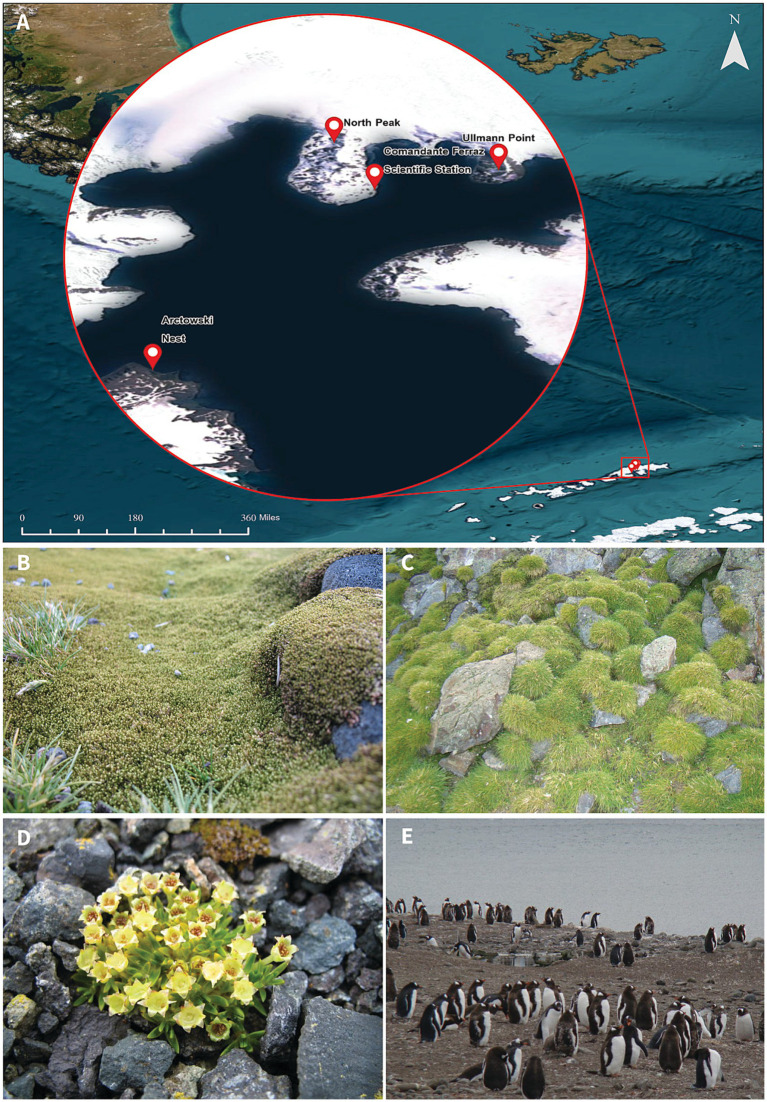
Sample sites at King George Island, Antarctic Peninsula. The island is part of the South Shetland Islands. **(A)** Map created using ArcGIS Pro v.3.2. **(B)** *Sanionia uncinata*. **(C)** *Deschampsia antarctica*. **(D)** *Colobanthus quitensis*. **(E)** Ornithogenic soil near an Adelie penguin nest.

Soil samples were collected in four ice-free sites on King George Island (Antarctic Peninsula) during the austral summer of 2007 (Figure 1A). The sampling sites and collected samples include the following:

1. soil under *Sanionia uncinata* from the North Peak (62°04′849”S, 58°24′024”W; Figure 1B),
2. the rhizosphere of *Deschampsia antarctica* from Ullmann Point (62°05′015”S, 58°23′987”W; Figure 1C),
3. the rhizosphere of *Colobanthus quitensis* from Comandante Ferraz Scientific Station (62°05′06”S, 58°24′12”W; Figure 1D), and
4. ornithogenic soil near an Adelie penguin nest from Arctowski Polish Station (62°09′790”S, 58°29′687”W; Figure 1E).

The method described by da Silva et al. (2017) was used to isolate culturable bacterial fraction. A preliminary evaluation of the 16S gene *rrs* indicated that the isolates belong to the genus *Pseudomonas*. The isolates are part of the Antarctic culture collection at the Microbial Molecular Ecology Laboratory (Federal University of Rio de Janeiro, Brazil).

Twenty-five psychrotolerant isolates affiliated with the genus *Pseudomonas* were selected for this study. Of these, 13 (52%) were isolated from ornithogenic soil, followed by the rhizosphere of the native plants *C. quitensis* (*n* = 6; 24%) and *D. antarctica* (*n* = 4; 16%). Two isolates (8%) were isolated from soil covered by the moss *S. uncinata*. The highest temperature at which we observed growth was 28°C. For this reason, the optimal incubation was 28°C for 24 h (antimicrobial resistance phenotypic screening) to 48 h (DNA extraction).

### 2.2 Mass spectrometry MALDI-TOF

**Figure 2:**
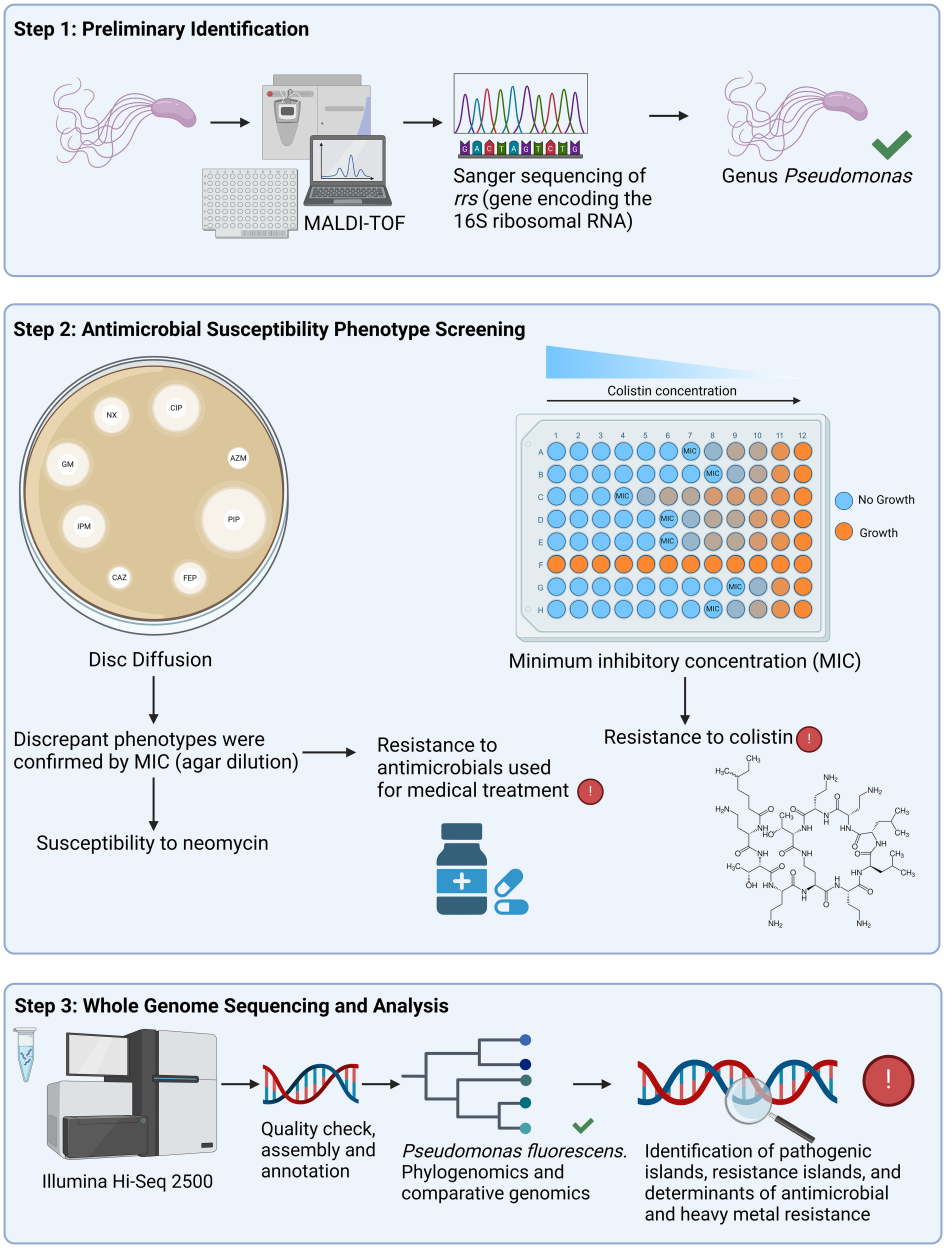
Schematic representation showing the division of the study in three steps. The first step was the preliminary identification using matrix-assisted laser desorption/ionization – time of flight (MALDI-TOF) and Sanger sequencing of the *rrs* gene, which encodes the 16S rRNA. Twenty-five isolates were confirmed to belong to the genus *Pseudomonas* and proceeded to the second step, which was the antimicrobial susceptibility screening. Antimicrobial agents were tested using disk diffusion, apart from colistin, which was analyzed with minimum inhibitory concentration (MIC) by broth microdilution [recommended by Clinical Laboratory Standards Institute (CLSI), 2023]. The isolates that displayed susceptibility to neomycin, and resistance to ceftazidime, cefepime and aztreonam were also evaluated with MIC by agar diffusion. The third step consisted of the whole genome sequencing (WGS) of fourteen non-clonal isolates, selected based on their resistance phenotypes. After the quality check and assembly of the raw sequences, we performed the phylogenomics and comparative genomics. Additionally, pathogenic and resistance islands, as well as antimicrobial and heavy metal resistance determinants were annotated. Created in BioRender. Silverio, M.P. https://BioRender.com/p48t351.

The genus of each isolate was confirmed using matrix-assisted laser desorption/ionization – time of flight (MALDI-TOF; Microflex LT, Bruker GmbH, Berlin, Germany). This experiment represented the beginning of the trial for *Pseudomonas* isolates (Figure 2), which was performed using the algorithm provided by the manufacturer. The colonies were transferred in triplicate to the plate “MSP 96 Polished Steel BC,” provided by the manufacturer. The plate was cleaned in accordance with the manufacturer’s instructions, with 70% alcohol followed by 80% trifluoroacetic acid. We added 1 μL of 70% formic acid (Tedia, Fairfield, Ohio, United States) and allowed the plate to fully dry at room temperature. Afterwards, 1 μL of the matrix *α*-cyano-4-hydroxycinnamic acid (Bruker GmbH, Berlin, Germany) diluted to 10 mg/mL in organic solvent [50% acetonitrile and 2.5% trifluoroacetic acid (Tedia, Fairfield, Ohio, United States)] was applied and dried at room temperature. The calibration strain was *Escherichia coli* ATCC 25922, which was the reference strain for the peaks in a spectrum of proteins between 2 and 20 kDa, as provided by the manufacturer (software FlexControl v.3.4, Bruker GmbH, Berlin, Germany). The results were compared with the spectra in MALDI Biotyper v 3.1, using the MBT Compass software and the MALDI Biotyper^®^ CA library (Bruker GmbH, Berlin, Germany).

### 2.3 DNA extraction, acid nucleic fingerprinting, and 16 S rRNA sequence analysis

The isolates were incubated at 28°C with constant shaking at 150 rpm, until the OD_600_ reached 1 (approximately 48 h). Bacterial genomic DNA was extracted using the Wizard Genomic DNA Purification Kit (Promega, Madison, Wisconsin, United States), following the manufacturer’s instructions. The DNA was quantified using a Qubit fluorometer (Invitrogen, Waltham, Massachusetts, United States) with the Qubit double-stranded DNA high sensitivity Assay Kit (Life Technologies, Carlsbad, California, United States).

First, the genetic diversity of the isolates was assessed via a repetitive extragenic palindromic polymerase chain reaction (BOX-PCR). The final concentration of each reagent was 1.0 μM of primer BOXA1-R CTACGGCAAGGCGACGCTGACG (Versalovic et al., 1994), 1.25 u of GoTaq^®^ G2 DNA polymerase, 1X Green GoTaq^®^ reaction buffer, 1.5 mM of MgCl_2_, 0.2 mM of deoxynucleotide triphosphate (Promega, Madison, Wisconsin, United States), and 50 ng/μL of genomic DNA with a final volume of 25 μL. The complete amplification cycle was one cycle of 95°C for 7 min, 30 cycles of 94°C for 1 min, 53°C for 1 min, 65°C for 8 min, and a final extension of one cycle at 65°C for 16 min in a thermocycler (Eppendorf, Hamburg, Germany). The products were analyzed using electrophoresis 1.5% agarose using the 1 kb DNA ladder (Thermo Fisher Scientific, Waltham, Massachusetts, United States). The run took place at 100 V for 30 min. The dendrograms were constructed using the program BioNumerics v.7, with default parameters (Biomérieux, Marcy-l’Étoile, France).

The amplification of the gene *rrs* was performed with 5 pmol/μL of each primer (27f AGAGTTTGATCATGGCTCAG and 1492r GTTTACCTTGTTACGACT) and a final fragment size of 1,465 base pairs (bp) (Lane, 1991). The reaction had a final volume of 50 μL, using the same concentrations of Taq, reaction buffer, MgCl_2_, deoxynucleotide triphosphate, and genomic DNA described above. The cycle was performed as follows: one cycle of 94°C for 3 min, 35 cycles of 94°C for 40 s, 55°C for 1 min, 72°C for 2 min, and a final extension of one cycle at 72°C for 10 min (Lane, 1991). The PCR products were analyzed as described above.

Additionally, each amplicon was purified with the enzyme ExoSAP (Exonuclease I, Shrimp Alkaline Phosphatase; Thermo Fisher Scientific, Waltham, Massachusetts, United States). The enzyme was diluted to a ratio of 1:9 in nuclease-free water (Qiagen, Hilden, Germany). Next, the following cycle was performed: one cycle of 37°C for 15 min (enzymatic optimal temperature) and one cycle of 80°C for 15 min (denaturation). In addition, 5 μL of pure amplicon and 5 μL of each primer (at a final concentration of 5 pmol/μL) were inoculated in a microplate and submitted for Sanger sequencing (Macrogen, Seoul, South Korea). The sequences were evaluated, trimmed and aligned using BioEdit v.7.2 (Hall, 1999). The species were defined using the tool Sequence Match, with the nonparametric *k*-nearest neighbors’ method, available at the Ribosomal Database Project (http://rdp.cme.msu.edu, accessed on March 15, 2019).

### 2.4 Antimicrobial susceptibility tests

Antimicrobial susceptibility was assessed using the disk diffusion method, according to the protocol M02 established by the Clinical Laboratory Standards Institute (CLSI) (2012a). The tests were performed with piperacillin (PIP, 100 μg), aztreonam (AZM, 30 μg), piperacillin-tazobactam (TZP, 110 μg), ceftazidime (CAZ, 30 μg), cefepime (FEP, 30 μg), imipenem (IPM, 10 μg), gentamicin (GM, 10 μg), norfloxacin (10 μg) and ciprofloxacin (5 μg). To check the evolutionary aspects, we also tested antimicrobials known to be ineffective against *P. aeruginosa*. The list included tetracycline (TE, 30 μg), sulfamethoxazole-trimethoprim (25 μg), NEO (30 μg), chloramphenicol (C, 30 μg), ertapenem (ETP, 10 μg), ampicillin (AM, 10 μg), amoxicillin-clavulanate (AMC, 30 μg), cephalothin (CF, 30 μg), and cefotaxime (CTX, 30 μg). These antimicrobials were selected to evaluate whether the Antarctic *P. fluorescens* isolates exhibited similar resistance patterns to *P. aeruginosa*.

To evaluate extended-spectrum beta-lactamase (ESBL) phenotypes, PIP, AZM, CAZ, and FEP disks were positioned 2.5 cm from TZP, while AM, CF, and CTX disks were placed 2.0 cm from AMC. The antimicrobial disks represented the product “sensifar” and were commercially obtained from Cefar (São Paulo, Brazil), except PIP, which was prepared using the lyophilized drug from MilliporeSigma (Burlington, Massachusetts, United States). In addition, *P. aeruginosa* ATCC 27853 was the positive control strain, and the data were interpreted using CLSI M100 [Clinical Laboratory Standards Institute (CLSI), 2023].

The minimum inhibitory concentration (MIC) of colistin (CL; MilliporeSigma, Burlington, Massachusetts, United States) was accessed using broth microdilution. Each 0.5 McFarland suspension was diluted with a cation-adjusted medium (0.2 mL of Ca^2+^ and 0.1 mL of Mg^2+^ to each 100 mL of Mueller Hinton; Difco Laboratories Inc., Detroit, Michigan, United States) [Clinical Laboratory Standards Institute (CLSI), 2012b]. Serial dilutions of CL, with concentrations between 0.032 and 256 μg/mL, were evaluated. The strains *P. aeruginosa* ATCC 27853 (MIC 0.5–4 μg/mL) and *E. coli* ATCC 25922 (MIC 0.25–2 μg/mL) were used as susceptible controls, and *E. coli* C153 (carrying *mcr-1*, MIC 8 μg/mL) was employed as the CL-resistant control.

The MIC of the beta-lactams CAZ, FEP, AZM, and aminoglycoside NEO (MilliporeSigma, Brulington, Massachusetts, United States) were analyzed using agar dilution [Clinical Laboratory Standards Institute (CLSI), 2012b]. The beta-lactams were dissolved and diluted according to the method in previous work [Clinical Laboratory Standards Institute (CLSI), 2023]. Moreover, NEO was dissolved and diluted with sterile distilled water to a final concentration of 50 mg/mL, following the manufacturer’s instructions. Serial plates with concentrations varying between 1 and 256 μg/mL were analyzed, and *P. aeruginosa* ATCC 27853 was the control. The results were interpreted using the MIC breakpoints for other non-*Enterobacterales* [Clinical Laboratory Standards Institute (CLSI), 2023]. When the breakpoints for other non-*Enterobacterales* were unavailable (as in the case of CL), *P. aeruginosa* breakpoints were used.

### 2.5 Whole genome sequencing, assembly, and annotation

Fourteen non-clonal isolates were selected for WGS. The selection criteria aimed to include isolates with high-level resistance phenotypes toward beta-lactams (specifically CAZ and FEP) and CL, and those with the most susceptible profiles.

Paired-end sequencing libraries (2×150 bp; 450 bp insert size) were constructed using 5 μg/μl of genomic DNA, following the NEBNext Fast DNA Fragmentation and Library Preparation Kit (New England Biolabs Inc., Ipswich, Massachusetts, United States). The quality control analysis of the final libraries was performed using the 2100 bioanalyzer (Agilent Technologies, Santa Clara, California, United States) and was visualized using electrophoresis 1.2% agarose. All samples were sequenced on the Illumina Hi-Seq 2500 platform (Illumina, San Diego, California, United States).

The quality of raw sequences was evaluated with FastQC v.0.11.5 (Andrews, 2010), and the reads and adaptors were trimmed using fastp v.0.23.4 (Chen et al., 2018) with default quality filter of >Q15. The genomes were assembled using Unicycler v.0.5.0 (Wick et al., 2017) with tested k-mer sizes of 27,53,71,87,99,111,119,127. The quality assessment of each assembly was checked with QUAST v.5.2.0 (Gurevich et al., 2013), CheckM2 v.1.0.2 (Chklovski et al., 2024) and GUNC v.1.0.2. As a quality filter, we considered N50 > 70 Kb (QUAST), completeness >90% (CheckM2), contamination <5% (CheckM2) and clade separation score > 0.45 (no chimeric contig) (GUNC). MOB-suite (Robertson and Nash, 2018) was used to identify plasmids in the draft genomes. The plasmid database used is available online at: https://zenodo.org/records/10304948/files/data.tar.gz, accessed on August 25, 2024. Default parameters were used for each software.

To determine the taxonomic classification of each *Pseudomonas* strain based on their genomes, we performed an analysis using the Genome Taxonomy Database Toolkit (GTDB-Tk) v.2.3.2 (Chaumeil et al., 2022) with the Classify workflow (“classify_wf”) and database r214. In the “ani_screen’ step it uses Mash v.2.3 (Ondov et al., 2016) to find the best hits among the representative genomes in the r214 database, then FastANI v.1.32 (Jain et al., 2018) to identify the species of the query genome using ≥95% as Average Nucleotide Identity (ANI) cutoff (Arahal, 2014). If the ANI analysis does not identify the query genome species the next steps are performed. In the “identify” step it employs Prodigal v.2.6.3 (Hyatt et al., 2010) and HMMER v.3.4 (Finn et al., 2011; Finn et al., 2015) for the identification of 120 bacterial phylogenetic marker genes and performs a multiple sequence alignment. The “align” step concatenates and filters the alignment. Finally, the “classify” step uses pplacer v1.1.alpha19-0-g807f6f3 (Matsen et al., 2010) to determine the place of the genome in the GTDB-Tk reference tree. The genomes were annotated using Prokka v.1.14.6 (Seemann, 2014).

### 2.6 Phylogenomic and comparative genomic analysis

The average nucleotide identity (ANIb) based on Basic Local Alignment Search Tool+ (BLAST+) and the correlation indices of tetra-nucleotide (TETRA) signatures of all analyzed genomes was run using default parameters on Pyani (Pritchard et al., 2016). The results were combined to assess the relationship between the genomes using the R package v.4.0.3, using the Euclidean distance and *dist* function from the statistics package for distance calculations. The *hclust* function from the statistics package was applied, using the average method for clustering calculations. Further details are provided in the online manuals (available at: https://www.rdocumentation.org/packages/stats/versions/3.6.2/topics/dist and https://www.rdocumentation.org/packages/stats/versions/3.6.2/topics/hclust, accessed on March 10, 2024).

A scatterplot of the correlation of ANIb and TETRA values was generated using the ggplot2 package (Wickham, 2016), and the correlation was evaluated using Spearman’s correlation and the *cor.test* function from the statistics package. The *cor.test* calculates an exact *p*-value when using “*cor.test* (clusteredAni$height, clusteredTetra$height, method = “spearman”).” The *shapiro.test* function from the statistics package was applied to assess the normality of the data, revealing a Gaussian distribution. The *shapiro.test* calculates an approximate *p*-value when using “*shapiro.test* (clusteredAni$height) and *shapiro.test* (clusteredTetra$height).” Further details are provided in the online manuals (available at: https://www.rdocumentation.org/packages/stats/versions/3.6.2/topics/shapiro.test and https://www.rdocumentation.org/packages/stats/versions/3.6.2/topics/cor.test, accessed on March 10, 2024). Additionally, the confidence of each clade was calculated with a bootstrap of 100 replicates using *pvclust 2.2–0* (Suzuki and Shimodaira, 2006), and the factoextra package was employed to generate the dendrogram. For the visualization of the graphs,

We ran Benchmarking Universal Single-Copy Orthologs (BUSCO) (Simao et al., 2015) and BUSCO Phylogenomics (Waterhouse et al., 2018) to create the supermatrix. Besides, MAFFT (Kuraku et al., 2013) was employed to align the sequences of the supermatrix. We used the “--auto” option from MAFFT, which selects the appropriate alignment strategy amongst FFT-NS-2, FFT-NS-i and L-INS-I, according to the size of the input data. Gblocks (Talavera and Castresana, 2007) extracted the best-aligned blocks, using “sequence type equals protein (−t = p)” and “minimum length of a block equals 5 (−b4 = 5)” as the parameters. The output was converted to the Phylogeny Inference Package (PHYLIP) format using ClustalW2 (Larkin et al., 2007). The phylogenomic analysis was performed using RAxML (Stamatakis, 2014), using 100 bootstrap repetitions. The substitution model was defined by “-mPROTGAMMAWAG,” in which the model of heterogeneity is “GAMMA” and the substitution model is “LG.” Further details are provided in the online manual (available at: https://cme.h-its.org/exelixis/resource/download/NewManual.pdf, accessed on March 10, 2024). The tree was visualized and colored using iTOL v.7 (Letunic and Bork, 2024).

Genome Unclutterer (GUNC) v1.0.6 (Orakov et al., 2021) with clade separation score (CSS) > 0.45 was used to detect chimeras, contamination and the annotation of plasmids. For the taxonomy curation, type strain genome server (available at Type Strain Genome Server (dsmz.de); accessed on March 23, 2024) and GTDB-Tk v2 were employed (Chaumeil et al., 2022).

### 2.7 Genomic analysis of *Pseudomonas fluorescens* regarding antimicrobial susceptibility

Antimicrobial resistance and further genes of interest were identified using the Genome Annotation tool available at the Pathosystems Resource Integration Center (PATRIC; available at Bacterial and Viral Bioinformatics Resource Center | BV-BRC, accessed on June 24, 2024) (Davis et al., 2020). For the annotation of ARGs, we applied the keywords “resistance,” “beta-lactam,” “penicillin,” “aminoglycoside,” “*aph*,” “*aac*,” “chloramphenicol,” “*cat*,” “polymyxin,” “*arn*,” “*pmrK*,” “tetracycline,” “*tetR*,” “efflux,” “ABC transporter,” and “ABC-type” using default parameters for Bacteria/Archaea. “Isopenicillin N epimerase” was detected for most of the isolates, but it was not included due to its importance on the antimicrobial biosynthesis pathway (not described in this work).

The keywords “heavy metal,” “arsenic” and “prophage” were also investigated. Relevant genes for the adaptation to extreme environments were searched and the function was manually assigned based on the annotation (Wattam et al., 2017).

The following databases were searched for ARGs using the ABRicate v.1.0.1 Pipeline: MEGARes v.3.0 (6635 sequences) (Lakin et al., 2017; Bonin et al., 2023), ResFinder v.4.1 (3077 sequences) (Bortolaia et al., 2020; Florensa et al., 2022), NCBI AMRFinderPlus v3.12.8 (5386 sequences) (Feldgarden et al., 2021; Feldgarden et al., 2022), ARG-ANNOT (2223 sequences) (Gupta et al., 2014), and CARD v.3.2.4 (2631 sequences) (Alcock et al., 2023; McArthur et al., 2013). We used ABRicate v.1.0.1 with a minimum identity threshold of 80% and a minimum coverage threshold of 80%. Databases were queried sequentially and overlapping predictions from multiple databases were considered a validation of results and retained in the final analysis. ABRicate was run on the Galaxy version 24.1.4.dev0 server, where its user-friendly interface allows for parameter configuration and database selection without requiring complex workflows. All databases (DbType nucI) were last updated November 4, 2023.

### 2.8 Prediction of pathogenicity and resistance islands

This study employed the Genomic Island Prediction Software (GIPSy) to check for genomic island availability (Soares et al., 2016). GIPSy uses an eight-step workflow for genomic island prediction, with each step incorporating specific default parameters to identify genomic features: Step 1 processes input files; Step 2 applies a G + C content deviation cutoff of 1.5 standard deviations; Step 3 uses a sensitivity setting of 0.95 for codon usage deviation (Colombo/SigiHMM); Step 4 predicts transposase genes with an HMMer e-value of 0.0001; Step 5 detects virulence or resistance factors using BLASTP with an e-value of 0.000001; Step 6 performs reciprocal BLAST with an e-value of 0.000001; Step 7 identifies tRNA flanking regions with an HMMer e-value of 0.0001; and Step 8 combines results from all previous steps to predict pathogenicity and resistance islands. All steps were executed using default settings provided by the software. In cases where regions were associated with more than one type of genomic island (pathogenic, metabolic, resistance, or symbiotic), PAIs and RIs were retained simultaneously, with overlapping regions plotted together at the same locus on the circular genomic graphs to reflect their dual classification. This research applied BLAST Ring Image Generation (Alikhan et al., 2011) to represent and evaluate the position of genomic islands in various strains of *Pseudomonas*, and the similarity between strains of the same species. The strain *P. antarctica* LMG 22709 (NZ_LT629704.1) was selected as the reference genome for GIPSy Island predictions. The strain *P. carnis* BML-PP010 (BQHE01000001.1) was applied as a reference for pathogenic strain. Genomic ring images were generated, and for groups with more than one sample, the largest genome was selected as the central ring for the circular plots.

### 2.9 Data availability

The *rrs* sequences encoding the 16S gene are provided under the nucleotide accession numbers MK681799.1 to MK681824.1. The complete genome sequence data, including raw sequence reads, genome assemblies, and annotations of Pseudomonads applied in this study, were submitted to GenBank under the BioProject accession PRJNA1183857. Supplementary Table S2 displays the nucleotide accession numbers of the isolates evaluated using phylogenomic analysis.

## 3 Results

### 3.1 Antimicrobial susceptibility screening

A screening with six distinct classes of antimicrobials was performed using the disk diffusion method in accordance with CLSI standards [Clinical Laboratory Standards Institute (CLSI), 2012a; Clinical Laboratory Standards Institute (CLSI), 2023]. Similar to the control strain *P. aeruginosa* ATCC 27853, the 25 isolates presented resistance to representatives of four classes of antimicrobials: C, sulfamethoxazole-trimethoprim, TE, and beta-lactams (AM, AMC, CTX, CF, and ETP). The evaluated isolates in this work did not present a halo distortion, suggesting the absence of ESBL.

All isolates presented a putative phenotype susceptible to NEO. The diameter of the inhibition zones varied between 19 and 26 mm, in contrast to the control strain (no observed halo).

Regarding the beta-lactams with breakpoints available for the clinical treatment of other non-Enterobacterales, all isolates presented phenotypes intermediate or resistant to AZM. Moreover, 11 isolates (50%) were intermediate/resistant to CAZ, six (25%) to FEP and one (4%) to IPM (Supplementary Table S1).

The MIC by agar dilution confirmed the low susceptibility to AZM, CAZ, and FEP, with MICs varying from 32 to ≥256 μg/mL, whereas *P. aeruginosa* ATCC 27853 displayed an MIC of 4 μg/mL for CAZ and AZM and 8 μg/mL for FEP. This method also confirmed the susceptibility of the Antarctic isolates to NEO, with MICs ranging from 2 to 8 μg/mL, whereas the control strain *P. aeruginosa* ATCC 27853 presented an MIC ≥256 μg/mL.

As stated by Clinical Laboratory Standards Institute (CLSI) (2023), an MIC ≤2 μg/mL is considered intermediate to CL, and a value ≥4 μg/mL is considered resistant. Likewise, all 25 isolates described in this work were intermediate or resistant to this polymyxin. The MIC was 0.5 μg/mL for four isolates, 1 μg/mL for seven, 2 μg/mL for two, and 4 μg/mL for one. Eleven isolates (44%) displayed higher MICs than the positive control *E. coli* C153 (16 μg/mL): two were 128 μg/mL, and nine were ≥ 256 μg/mL. Table 1 presents the phenotypic data for 14 isolates selected for WGS. Supplementary Table S1 presents the complete data for the 25 phenotypically analyzed isolates.

**Table 1:** Antimicrobial resistance profiles of 14 *Pseudomonas* sp. strains accessed using disk diffusion, with minimal inhibitory concentrations (MICs) for selected antimicrobial agents.

Strains	Antimicrobial susceptibility testing results in mm (MIC in μg/mL)																		
	AM	AMC	CF	CTX	ETP	SXT	NEO	C	TE	PIP	TZP	CAZ	FEP	AZM	IPM	GM	CIP	NX	CL
O11	6	6	6	16	8	6	20 (8)	18	28	31	33	**14 (128)**	**14 (128)**	**6 (128)**	25	26	33	32	**(≥256)**
O39	6	6	6	27	11	21	23 (2)	12	30	37	38	30	22	**10 (128)**	20	27	34	34	**(128)**
D47	6	6	6	14	14	15	21 (4)	10	25	32	35	**6 (128)**	**6 (128)**	**6 (128)**	26	27	35	36	**(≥256)**
O62	6	6	6	6	6	16	23 (4)	15	28	30	24	**6 (≥256)**	**8 (≥256)**	**6 (128)**	**16**	36	34	32	**(≥256)**
O64	6	6	6	6	6	6	26 (4)	17	39	36	38	20	**16 (64)**	**6 (128)**	22	30	39	36	**(≥256)**
S101	6	6	6	6	14	6	24 (4)	13	23	33	34	**6 (≥256)**	23	**6 (128)**	30	27	37	36	**(≥256)**
D118	6	6	6	6	20	9	22 (4)	11	25	31	30	22	20	**6 (≥256)**	35	28	40	35	**(2)**
O160	6	12	6	11	23	6	22 (2)	6	25	30	30	**12 (64)**	26	**6 (128)**	34	24	39	36	**(0.5)**
S191	6	6	6	6	12	6	21 (4)	16	27	31	32	**17 (≥256)**	18	**6 (128)**	27	27	38	32	**(≥256)**
O230	6	6	6	24	37	6	21 (4)	18	32	30	32	27	28	**6 (128)**	38	22	40	38	**(1)**
D277	6	6	6	11	13	6	22 (8)	10	28	30	29	23	23	**6 (128)**	25	27	37	34	**(2)**
C290	6	6	6	9	13	6	20 (4)	16	21	29	30	22	26	**6 (128)**	30	25	40	36	**(4)**
C291	6	6	6	9	16	11	22 (8)	9	22	32	39	22 (32)	22	**6 (128)**	30	25	40	34	**(1)**
O329	6	6	6	6	12	6	20 (4)	6	30	31	31	25	25	**6 (128)**	32	23	33	32	**(1)**
*P. aeruginosa* ATCC 27853	6	6	6	24	25	6	6 (≥8)	6	23	33	32	30 (4)	30 (8)	30 (4)	34	25	28	24	(2)
*E. coli* ATCC 25922	-	-		-	-	-	- (4)	-	-	-	-.	-	-	-	-	-	-	-	(1)
*E. coli* C153		-		-	-	-	-	-	-	-	-	-	-	-	-	-	-	-	(8)

In bold: intermediate/resistant phenotype against antimicrobials available for clinical treatment of Pseudomonas. AM: ampicillin; AMC: amoxicillin-clavulanate; CF: cephalothin; CTX: cefotaxime; ETP: ertapenem; SXT: sulfamethoxazole-trimethoprim; NEO: neomycin; C: chloramphenicol; TE: tetracycline; PIP: piperacillin; TZP: piperacillin-tazobactam; CAZ: ceftazidime; FEP: cefepime; AZM: aztreonam; IPM: imipenem; GM: gentamicin; CIP: ciprofloxacin; NX: norfloxacin; CL: colistin; − not determined.

### 3.2 Genomic characterization of *Pseudomonas fluorescens*

The MALDI-TOF analysis (scores between 1,703 and 2,259) confirmed that the isolates belong to the genus *Pseudomonas*. According to BOX-PCR, seven clusters ranged from two to four isolates, represented by clones (Supplementary Figure S1). Isolates representative of such genetic diversity or displaying diverse antimicrobial susceptibility profiles were selected for WGS.

The average genome size of the *Pseudomonas* was 6.5 Mb (varying between 5.8 and 7.6 Mb) and presented an average GC ratio of 59.7% (between 58 and 60%). In addition, an average of 6,135,071 protein coding sequences were identified. Integrated prophages were found across each genome, in which the most common encoded protein was Gifsy-2, followed by an antirepressor, CP4-57 regulatory, and Lp2 protein 6. Table 2 summarizes the general features of the draft genomes of the *Pseudomonas* isolates.

**Table 2:** Genome features of 14 strains isolated from Antarctic samples submitted to whole gene sequencing.

Strain	Genome size (bp)	GC content (%)	CDS	rRNA	tRNA	Number of contigs	Coverage	Accession number
O11	6,546,509	59.85	5,957	2	60	187	584	JBJGXW000000000
O39	6,325,140	59.77	5,745	3	61	101	837	JBJGXV000000000
D47	6,881,600	59.58	6,254	2	58	236	307	JBJGXU000000000
O62	6,521,202	59.81	5,986	2	60	187	299	JBJGXT000000000
O64	6,515,294	59.81	5,920	2	54	195	353	JBJGXS000000000
S101	6,518,967	59.43	5,967	2	61	239	353	JBJGXR000000000
D118	7,671,351	59.89	71,141	2	54	257	263	JBJGXQ000000000
O160	5,892,666	59.74	5,168	3	60	59	414	JBJGXP000000000
S191	6,551,480	59.81	6,029	2	60	155	302	JBJGXO000000000
O230	6,428,734	58.95	5,999	4	61	34	328	JBJGXN000000000
D277	6,045,863	60.04	5,530	4	61	36	362	JBJGXM000000000
C290	6,297,686	59.86	5,818	2	61	49	456	JBJGXL000000000
C291	6,586,429	59.6	6,165	2	47	78	301	JBJGXK000000000
O329	6,262,247	60.23	5,724	2	55	58	270	JBJGXJ000000000

bp: base pairs.

According to the phylogenomic analyses based on the average nucleotide identity (ANI) derived from the complete genome distance matrix and the correlation of tetra-nucleotide frequency (TETRA), the *P. fluorescens* isolates from Antarctic samples exhibited high similarity to each other (ANIb >98%). The isolates belong to the *P. fluorescens* group, forming two distinct clusters (Supplementary Figure S2). The first and largest cluster comprised the isolates C291, O329, and O62 from a recent common ancestor, whereas C290, S191, S101, D118, and O160 were distant from each other. In contrast, the second cluster displayed less divergence between the isolates O230, O39, D277, O11, D47, and O64. Moreover, ANIb and TETRA varied between 0.0 and 0.3, with an outlier after 0.4.

**Figure 3:**
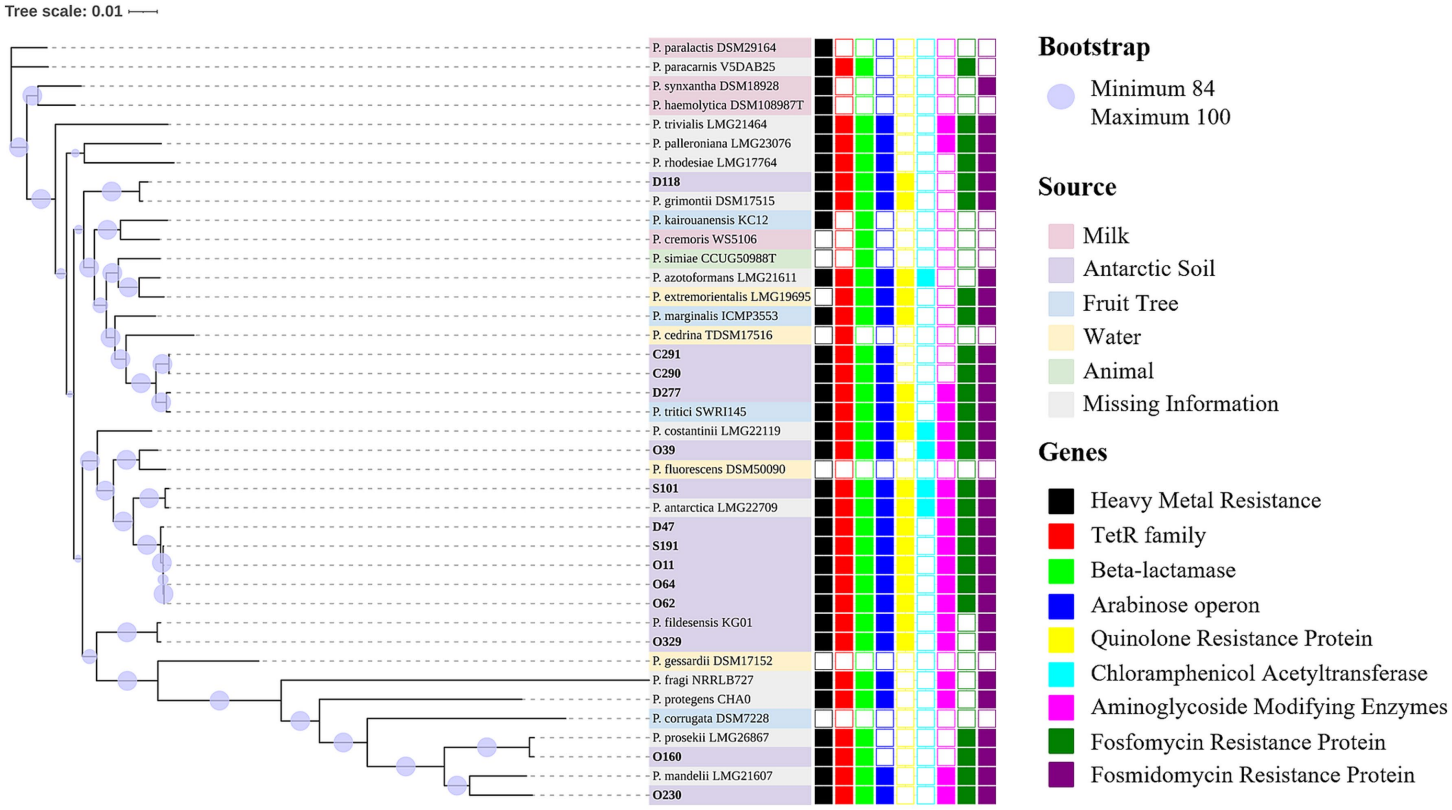
Phylogenomic analysis of Antarctic *Pseudomonas fluorescens* isolates (in bold). Bootstrap values range from 84 to 100 and are displayed as purple circles. Three potential new species were identified. The first cluster of potential new species isolates was D47, O62, O64, O11, and S191. The second comprises isolate O39, with the closest similarity to *P. fluorescens* DSM 50090. The third was O230, which is closest to *P. mandelii* LMG 21607. Regarding previously described species, S101 was identified as *P. antarctica* and is close to *P. antarctica* LGM 22709. Strain D118 was identified as *P. grimontii*, which is close to *P. grimontii* DSM 17515. Isolate O160 was identified as *P. prosekii* similar to *P. prosekii* LMG 26867, whereas O329 (*P. fildesensis*) is similar to *P. fildesensis* KG01. The Antarctic isolates D277, C290, and C291 belong to the species *P. tritici* and formed a cluster with *P. tritici* SWRI 145. Supplementary Table S2 presents the nucleotide accession numbers of the reference strains. The source is indicated by the label colors: milk (pink), Antarctic soil (purple), fruit tree (blue), water (yellow), animal (green) and missing information (grey). Gene annotation is displayed as squares. The full squares display the presence of the genes, while the empty squares show the absence. Resistance genes were identified for heavy metal (black), tetracycline (red), beta-lactamase (lime), colistin (operon *arn*; blue), quinolone (yellow), chloramphenicol (cyan), aminoglycoside (magenta), fosfomycin (green) and fosmidomycin (purple). Additionally, genes encoding efflux pumps were frequently detected in large quantities. Genes of adaptation to extreme conditions were detected in each isolate.

The taxonomy was curated using GTDB-Tk (Chaumeil et al., 2022) and the Type Strain Genome Server, and all isolates belong to the *P. fluorescens* group. In general, three potential new species were identified (Figure 3). Apart from O62 and O64, presenting a completeness of 99.99%, all genomes presented a completeness of 100%. Furthermore, all presented a very low contamination ratio [between 0 (C290) and 1.49 (D118)], indicating that contaminant contigs from other genomes were not identified.

### 3.3 Antimicrobial resistance genes in *Pseudomonas fluorescens* genomes

This study investigated the genes potentially involved in TE (*tetR*), aminoglycoside [*aph(3′)* and *aac(6′)*], polymyxin (*arnC, D, E, F*), fosfomycin (*fosA*) and, to a lesser extent, C (*cat*). Unknown quinolone and fosmidomycin resistance proteins were also detected. The quinolone resistance protein, which could possibly confer resistance to the first generation (i.e., nalidixic acid; not assayed in this work), was identified in nine isolates and presented 100% coverage and over 99% identity with other *P. fluorescens* isolates. The penicillinases penicillin-insensitive transglycosylase (EC 2.4.2.-), transpeptidase penicillin-binding protein (PBP-1C), other PBPs, class C beta-lactamases (EC 3.5.2.6) and metallo-beta-lactamases were frequently identified (Figure 3; Table 3).

**Table 3:** Description of *Pseudomonas fluorescens* spp. and annotation of relevant pathogenicity islands (PAIs), resistance islands (RIs), antimicrobial resistance genes (ARGs), and prophages.

Strains	Species	PAIs	RIs	Total	ARGs
O11	PNS.1	11	7	18	*tetR*, *arnC, D, E, F*, *fosA*, *aph*, aac, beta-lactamases*, “quinolone resistance protein,” “fosmidomycin resistance protein”
O39	PNS.3	10	8	18	*tetR*, *arnDEF*, *fosA*, *aph*, *aac*, *cat*, beta-lactamases*, “fosmidomycin resistance protein”
D47	PNS.1	22	7	29	*tetR*, *arnDEF*, *fosA*, *aph*, beta-lactamases*, “quinolone resistance protein,” “fosmidomycin resistance protein”
O62	PNS.1	13	8	21	*tetR*, *arnDEF*, *fosA*, *aph*, beta-lactamases*, “quinolone resistance protein,” “fosmidomycin resistance protein”
O64	PNS.1	13	7	20	*tetR*, *arnDEF*, *fosA*, *aph*, beta-lactamases*, “quinolone resistance protein,” “fosmidomycin resistance protein”
S101	*P. antarctica*	10	1	11	*tetR*, *arnDEF*, *fosA*, *aph*, *cat*, beta-lactamases*, “quinolone resistance protein,” “fosmidomycin resistance protein”
D118	*P. grimontii*	N.D.	N.D.	N.D.	*tetR*, *arnCDEF*, *fosA*, beta-lactamases*, “quinolone resistance protein,” “fosmidomycin resistance protein”
O160	*P. prosekii*	9	8	17	*tetR*, *arnDEF*, *fosA*, beta-lactamases*, “fosmidomycin resistance protein”
S191	PNS.1	11	8	19	*tetR*, *arnDEF*, *fosA*, *aph*, beta-lactamases*, “quinolone resistance protein,” “fosmidomycin resistance protein”
O230	PNS.2	10	9	19	*tetR*, *arnDEF*, *fosA*, *aac*, beta-lactamases*, “fosmidomycin resistance protein”
D277	*P. tritici*	8	7	15	*tetR*, *arnDEF*, *fosA*, *aph*, beta-lactamases*, “quinolone resistance protein,” “fosmidomycin resistance protein”
C290	*P. tritici*	10	6	16	*tetR*, *arnDEF*, *fosA*, beta-lactamases*, “fosmidomycin resistance protein”
C291	*P. tritici*	9	6	15	*tetR*, *arnDEF*, *fosA*, beta-lactamases*, “fosmidomycin resistance protein”
O329	*P. fildesensis*	8	9	17	*tetR*, *arnDEF*, *aac*, beta-lactamases*, “quinolone resistance protein,” “fosmidomycin resistance protein”

The reference strain *P. antarctica* LMG 22709 (NZ_LT629704.1) presented six PAIs and seven RIs. PNS: potential new species; ND: not detected. * includes penicillin-binding proteins. Efflux pumps from the superfamilies were detected (in order from high to low): ABC transporters, resistance nodulation division, major facilitator superfamily, multidrug and toxic compound extrusion, and small multidrug resistance. Heavy-metal resistance determinants, SOS response genes (recA, recX, and lexA), mismatch repair genes (mutL and mutS), osmotolerance gene (kdpD), tolerance to high or low-temperature genes (dnaK, dnaJ, grpE, groEL, groES, htpX, rpoH, cspD, and cshA), and phosphorous uptake optimization genes (pstA, pstB, and pstC) were detected in all isolates.

Hundreds of copies of genes encoding efflux pumps were predominantly found in each sample. In order of frequency, all five well-known efflux pump families were detected: ABC transporters (including the gene encoding the macrolide-specific efflux protein MacA), resistance nodulation division, major facilitator superfamily, multidrug and toxic compound extrusion, and small multidrug resistance proteins (Table 3; Supplementary Table S3). The overexpression of efflux pumps, alongside the activity of beta-lactamases, could be responsible for the observed phenotype of resistance towards AZM, CAZ and FEP. Further experiments with efflux pump inhibitors could help in the understanding of the resistance mechanism.

The beta-lactamase *bla*_PFM-2_ responsible for carbapenem resistance, which was identified in only one of the tested isolates (strain D47), was identified using NCBI AMRFinderPlus. In the databases CARD and MEGARes, only efflux pump genes from the resistance nodulation division family were detected (Supplementary Table S3). No ARGs were detected using the databases ResFinder and ARG-ANNOT. Moreover, only one plasmid was detected (isolate D118), which did not exhibit antimicrobial resistance determinants (Supplementary Figure S3). Overall, the data suggest that the variety of efflux pumps in the *P. fluorescens* genomes evaluated in this work plays a significant role in the observed antimicrobial resistance.

### 3.4 Identification of pathogenic and resistance islands

Apart from the isolate D118, PAIs and RIs were identified in all characterized genomes. The PAIs were more frequent, varying from six to 22, whereas up to nine RIs were identified. In general, the genomes described in this work presented higher numbers of PAIs and RIs than the pathogenic reference strain (*P. carnis* BML-PP010; BQHE01000001.1). The isolate D47 presented the highest total PAIs and RIs (*n* = 29), whereas S101 was even lower than the pathogenic reference strain (11 *versus* 13; Table 3). Supplementary Figure S4 presents the circular genome comparison plots displaying the islands identified against the genus *Pseudomonas*, reference strain *P. antarctica* LMG 22709 (NZ_LT629704.1) and pathogenic reference *Pseudomonas carnis* BML-PP010 (BQHE01000001.1).

### 3.5 Genes encoding heavy-metal resistance and adaptation to extreme environments

Copies of genes encoding the heavy-metal response regulator, heavy-metal sensor histidine kinase and heavy-metal resistance transcriptional regulator (*hmrR*) were identified in the genomes. One copy of a membrane-bound cytochrome biogenesis *cycz*-like domain, annotated as a heavy-metal associated domain, was identified in each genome. In addition, genes encoding arsenic resistance proteins were also found, varying from zero to five copies.

The DNA repair system genes (*recA*, *recX*, *mutL*, and *mutS*) responsible for the resistance to ionizing radiation were identified in each genome. Likewise, *lexA* (signaling and regulation) and *kdpD* (osmotolerance) were also observed. Genes conferring resistance to high pressure or high temperature (*dnaK*, *groEL*, *dnaJ*, *grpe*, *groES*, *htpX*, and *rpoH*) were also detected, and two genes responsible for the resistance to low temperatures (*cspD* and *cshA*) were found. The genes *pstA*, *pstB*, and *pstC* involved in the optimization of phosphorus uptake were detected. The frequency of these bacterial adaptation genes was generally low (from one to three copies), except for *dnaJ*, where the genomes presented three to five copies each.

## 4 Discussion

Horizontal gene transfer has played a critical role in the appearance of antimicrobial resistance in human-affected sites, which is sometimes also the case for so-called pristine environments. Previous studies have identified members of the *P. fluorescens* complex harboring ARGs in pristine or human/animal migration-affected areas in Antarctica (Orellana-Saez et al., 2019; Na et al., 2021; Marcoleta et al., 2022). Furthermore, genes conferring resistance to glycopeptides (*vanA/vanD* and *vanB*), methilicin (*mecA-*), and the New Delhi metallo-beta-lactamase (*bla*_NDM_) were recently identified in the animal feces of native Antarctic animals (Dimov and Strateva, 2022). Similarly, a study published in 2019 reported the presence of sulfonamide resistance genes (*sul1* and *sul2*) and a quinolone resistance gene (*qnrS*) in fecal samples collected from animals in the Fildes Peninsula, King George Island, Antarctica (Na et al., 2019). A positive correlation between *sul1* and *int1* was identified, suggesting that *int1* could be involved in spreading ARGs. Sellera and colaborators (Sellera et al., 2017) documented cases of migratory Magellanic penguins (*Spheniscus magellanicus*) found on the southeast coast of Brazil. These penguins, suffering from pododermatitis, carried *E. coli* with *mcr-1* and *bla*_ctx-m_ genes, which confer resistance to colistin and ESBLs, respectively. In the future, research focused on monitoring migratory animals could provide valuable insights into the evolution of antimicrobial resistance and the global dissemination of ARGs.

Antimicrobial resistance is often linked to metal resistance. Metal pollution reaches polar regions through atmospheric and oceanic circulation or through transport by migratory animals (Blais et al., 2005; Mechirackal Balan et al., 2018). Two mechanisms are known to drive the co-selection of metal and antimicrobial resistance. The first, known as “co-resistance,” involves metal- and antimicrobial-resistance determinants being encoded on the same mobile genetic element. The second, referred to as “cross-resistance,” occurs when a single mechanism, such as the overexpression of efflux pumps, confers resistance to both metals and antimicrobial agents (Henriques et al., 2016; Seiler and Berendonk, 2012). Previously, *P. frederiksbergensis* strain SS18 was highly resistant to mercury and to seven tested antimicrobials (unspecified) (Mechirackal Balan et al., 2018). The strain was isolated from Ny-Ålesund, Svalbard, Arctic, where coal was commercially exploited until the 1960s (Mechirackal Balan et al., 2018). Additionally, mercury and tellurite cross-resistance have previously been identified in three *Pseudomonas* isolates (Rodriguez-Rojas et al., 2016). The same isolates were resistant to nearly all tested antimicrobials (unspecified, but they were susceptible to amikacin, GM, and ciprofloxacin) (Rodriguez-Rojas et al., 2016).

Efflux pumps play a critical role in the extrusion of toxic compounds (Olivares Pacheco et al., 2017) and have been largely detected in Antarctic microbial isolates. For instance, *Pseudomonas* sp. strain MPC6, which was isolated from a soil sample on Deception Island (Antarctica), lacks genes encoding aminoglycoside-modifying enzymes, beta-lactamases and chloramphenicol acetyltransferases. Nevertheless, its genome carried genes encoding a variety of efflux pumps (Orellana-Saez et al., 2019). Previously, two *P. fluorescens* isolates carrying the efflux pump EmhABC presented resistance to C, nalidixic acid, AM, and TE (Hearn et al., 2003; Hearn et al., 2006; Tian et al., 2010). In this work, the observed C-resistant phenotype could be primarily due to the extrusion by efflux pumps, as *cat* genes were detected in only two of the isolates described here (Figure 3). Additionally, eleven isolates presented a phenotype intermediate/resistant to CAZ. Although this beta-lactam is considered for medical treatment against *Pseudomonas* infection [Clinical Laboratory Standards Institute (CLSI), 2023], resistance to CAZ in combination with avibactam was previously related to the presence of *bla*_VIM-1_ and *bla*_VIM-2_ and the overexpression of MexAB-OprM (Castanheira et al., 2019). Recently, Marcoleta et al. (2022) reported that two multidrug-resistant *P. fluorescens* isolates did not present ARGs in common with the reference strain *P. aeruginosa* PA7. Instead, these *P. fluorescens* isolates displayed a higher number of genes associated with ABC transporter and SMR efflux pumps (Marcoleta et al., 2022). Conducting functional assays on the activity of efflux pumps in Antarctic *P. fluorescens* will offer valuable insights.

In this study, the isolates displayed a multidrug-resistant phenotype, likely due to intrinsic features. The *tetR* gene, found in all of the isolates, is commonly found in the genus *Pseudomonas* because of its function of controlling the expression of genes involved in antimicrobial resistance and enzymes from catabolic pathways, the biosynthesis of antimicrobials, osmotic stress, and pathogenicity (Liu et al., 2013; Zhang et al., 2022; Shah and Sorum, 2014). The previous detection of the gene cluster *sul2*-*strA*-*strB* in ice cores from Dome Fuji Station (Eastern Antarctica) highlights the hypothesis that ARGs present distinct functions and may have existed before the preantimicrobial era (Okubo et al., 2019). Additionally, copies of genes encoded by the operon *arnBCADTEF* (previously known as *pmrHFIJKLM*), conferring polymyxin resistance in Gram-negative bacteria (Munoz-Escudero et al., 2023), were identified in most of the isolates (except O160, which presented an MIC of 0.5 μg/mL for CL). When the operon *arn* is induced, a 4-amino-4-deoxy-L-arabinose is added to the lipid A structure (Fernandez et al., 2010; Silverio et al., 2022; Moskowitz and Ernst, 2010; Munoz-Escudero et al., 2023). The isolates that represented a potential new species presented the highest observed MICs for CL (128 and ≥ 256 μg/mL; Table 1).

Pseudomonads are often intrinsically resistant to aminoglycosides due to chromosomal aminoglycoside-modifying enzymes (Zeng and Jin, 2003; Papapetropoulou et al., 1994). Although all isolates were susceptible to NEO, we identified aminoglycoside phosphotransferase [*aph(−3′)*] and aminoglycoside acetyltransferase [*aac-(6′)*] genes in most genomes (except for the isolates D118, O160, C290, and C291). The *aph(3′)* gene encodes phosphotransferases that confer resistance to NEO and kanamycin (Papapetropoulou et al., 1994; Zeng and Jin, 2003), while the *aac(6′)* gene encodes acetyltransferases that are active against a broad range of aminoglycosides, with the exception of gentamicin (GM) (Kawabe et al., 1975; Kobayashi et al., 2013). Furthermore, the phosphate uptake gene *pstB* was identified in all isolates, being previously linked to the intrinsic resistance of *P. aeruginosa* to aminoglycosides (Krahn et al., 2012). Although the isolates were susceptible to NEO, the lack of sequence homology prevented checking for gene mutations. Further transcriptomic analysis is necessary to investigate whether these genes are inactive.

The Antarctic *P. fluorescens* isolates exhibited resistance to AZM, an antimicrobial agent used in clinical treatment against *P. aeruginosa*. A previous study also identified AZM and carbapenem resistance in *P. fluorescens* isolates from chicken meat (Heir et al., 2021). While acquired beta-lactamase genes were absent in these isolates, the authors detected genes encoding efflux pumps, as well as *bla_AmpC_* and the PBP-encoding gene *mrcA*. Additionally, some isolates presented the gene *pbpC*, encoding a PBP3 homolog that might behave as a target for AZM (Heir et al., 2021; Jorth et al., 2017). In our study, we found that the Antarctic *P. fluorescens* isolates frequently harbor genes encoding various PBPs. Previous studies have reported that *P. fluorescens* isolates from pristine environments were resistant to several clinically important antimicrobial agents, including AZM, PIP, CAZ, CL, and various carbapenems (Pavlov et al., 2020; Poblete-Morales et al., 2020; Svec et al., 2020). One isolate, identified as *P. fildesensis*, was collected from Antarctic soil at the King George Island and exhibited genomic islands and other likely acquired mobile genetic elements (Pavlov et al., 2020). These findings underscore the potential pathogenicity of *P. fluorescens* isolates from remote environments.

The isolates presented not only a vast amount of efflux pumps, but also antimicrobial- and heavy-metal resistance genes (Figure 3; Table 3; Supplementary Table S3). The determinants *hmrR*, “DNA binding heavy-metal response regulator,” and “heavy-metal sensor histidine kinase,” alongside genes specifically related to arsenic resistance, were frequently identified. Previously, sodium arsenate and sodium arsenite intrinsic resistance were described in *P. fluorescens*, encoded by an operon with an arsenite inducible repressor (regulating the expression of arsenate reductase) and an ATP-dependent efflux pump (Prithivirajsingh et al., 2001). However, conducting functional assays to validate resistance to heavy metal is crucial.

When using genomic data for taxonomy assignments, the query genomes were compared to a database of type strains or reference genomes. The cutoff values for considering two genomes from the same species are ANI > 95% (Jain et al., 2018), dDDH >70% and < 1% divergence of G + C content (Meier-Kolthoff and Goker, 2019). Based on our results, all genomes are from the genus *Pseudomonas* (Supplementary Table S4). Within the genomes, the already described species are *P. antarctica* (S101), *P. fildesensis* (O329), *P. grimontii* (D118), *P. prosekii* (O160) and *P. tritici* (D277, C290 and C291). The novel species are PNS 1 (O11, D47, O62, O64 and S191), PNS 2 (O230), and PNS 3 (O39).

In this study, all of the described isolates belonged to the *Pseudomonas fluorescens* complex. Seven isolates derived from ornithogenic soil, five from the rhizosphere of native Antarctic plants (*Deschampsia antarctica* and *Colobanthus quitensis*) and two from soil beneath moss (*Sanionia uncinata*). One plasmid was detected, but it did not carry ARGs (*P. grimontii* D118; Supplementary Figure S2). The findings suggest that the observed antimicrobial-resistant phenotypes occurred due to intrinsic features. Nevertheless, we found a beta-lactamase gene encoding a PFM-like carbapenemase in one isolate (D47), which could pose a severe threat to clinical health. This PFM-like metallo-beta-lactamase was previously found in *P. synxantha* from chicken meat and was described by Poirel et al. (2020). The shared amino acid identity was over 90% for other species belonging to the *P. fluorescens* complex, indicating that this complex might function similarly to a reservoir (Poirel et al., 2020). Although the isolate did not present a discrepant phenotype when compared to the remaining Antarctic *P. fluorescens* isolates, further research based on transcriptomic and proteomic approaches need to be conducted, especially because the isolate D47 also presented the highest number of PAIs and RIs.

## 5 Conclusion

This study examines the evolutionary characteristics of antimicrobial resistance in *P. fluorescens* isolates from pristine environments in Antarctica. Resistance was observed to beta-lactams commonly used in clinical treatment, including aztreonam and ceftazidime, while resistance to cefepime and imipenem was detected to a lesser degree. Most of the isolates harbored genes typically considered intrinsic to the *Pseudomonas* genus, encoding promiscuous enzymes. Interestingly, despite the presence of aminoglycoside-modifying enzymes, the isolates remained susceptible to neomycin, indicating that the corresponding gene was likely inactive. Neomycin, an antimicrobial agent known to be ineffective against *P. aeruginosa*, was tested as part of an investigation into whether *P. fluorescens* from Antarctica would exhibit similar resistance patterns. Additionally, several copies of genes related to efflux pumps, heavy metal resistance, prophages, and adaptations to extreme environments were identified. These findings suggest that functional assays, transcriptomics, and proteomics would be crucial for further exploring the roles and functionality of these genes.

## Data Availability

The *rrs* sequences encoding the 16S gene are provided under the nucleotide accession numbers MK681799.1 to MK681824.1. The complete genome sequence data, including raw sequence reads, genome assemblies, and annotations of Pseudomonads applied in this study, were submitted to GenBank under the BioProject accession PRJNA1183857. Supplementary Table S2 displays the nucleotide accession numbers of the isolates evaluated using phylogenomic analysis.
