# Supplementary material for: Genomic and phenotypic insight into antimicrobial resistance of Pseudomonas fluorescens from King George Island, Antarctica
Source: Front Microbiol. 2025 Mar 3;16:1535420. doi: 10.3389/fmicb.2025.1535420 (PMC11911486; doi:10.3389/fmicb.2025.1535420)
Supplement: Supplementary file 1 [file Data_Sheet_1.docx]

Supplementary Material

# Supplementary Data

## Supplementary Figures


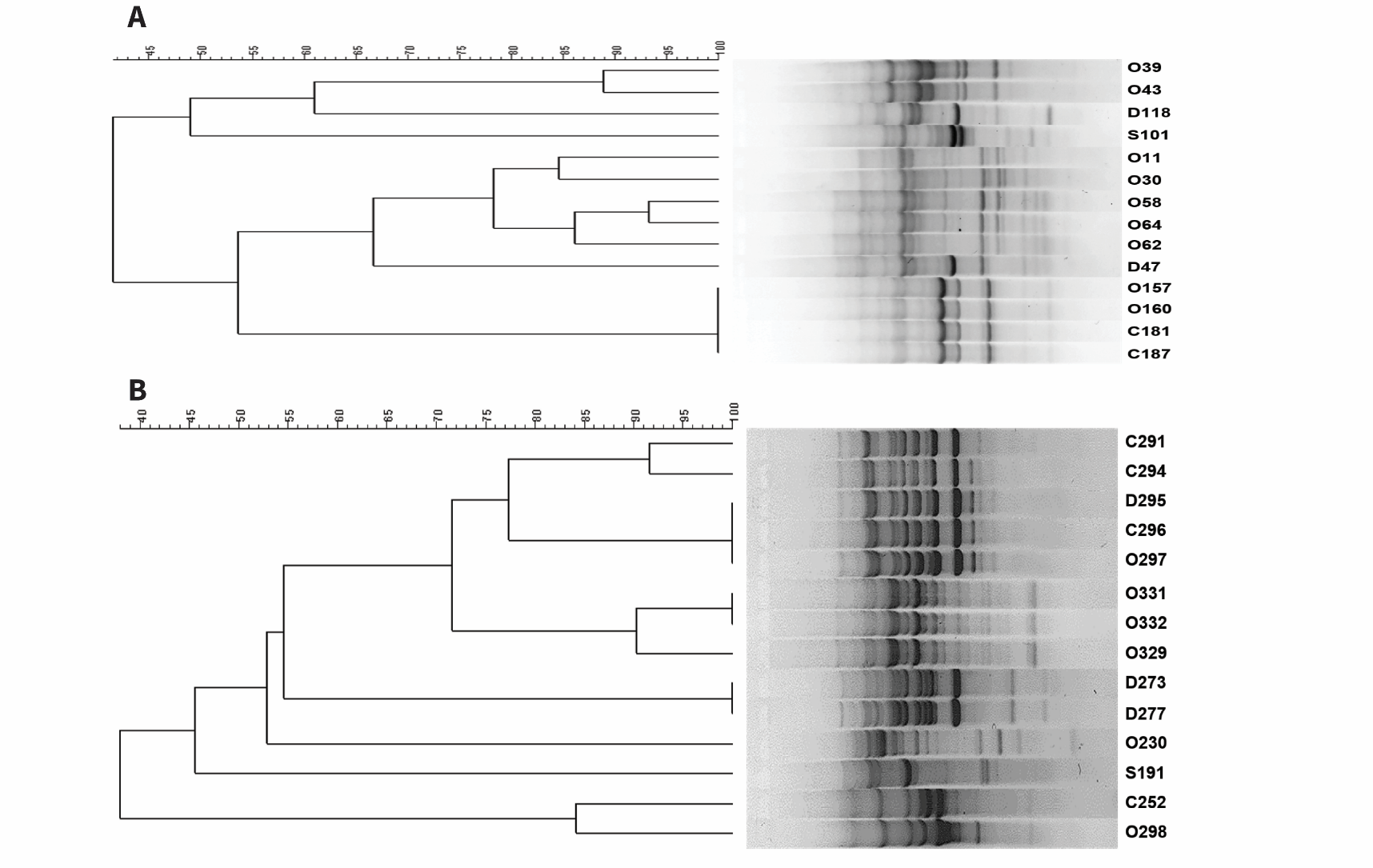
**Supplementary Figure 1: Dendrograms revealing the genetic diversity of members of the *Pseudomonas fluorescens* complex. A)** Isolates O39 and O43; O11 and O30; O58, O62, and O64; and O157, O160, C181, and C187 presented identical DNA patterns and were considered clones. **B)** Isolates C294, D295, C296, and O297; O331, O332, and O329; and D273 and D277 were considered clones. Isolate O298 was contaminated and not included. The phylogenomic analysis indicated that the isolates D295, C296, and O297 were classified as *Bacillus cereus* and were also not included. O: ornithogenic soil; C: *Colobanthus quitensis* rhizosphere; D: *Deschampsia antarctica* rhizosphere; S: *Sanionia uncinata* coverage soil. The dendrograms were constructed using BioNumerics v.7 (Biomérieux, Marcy-l’Étoile, France) and were employed as a cutoff on the strain submission to whole genome sequencing.


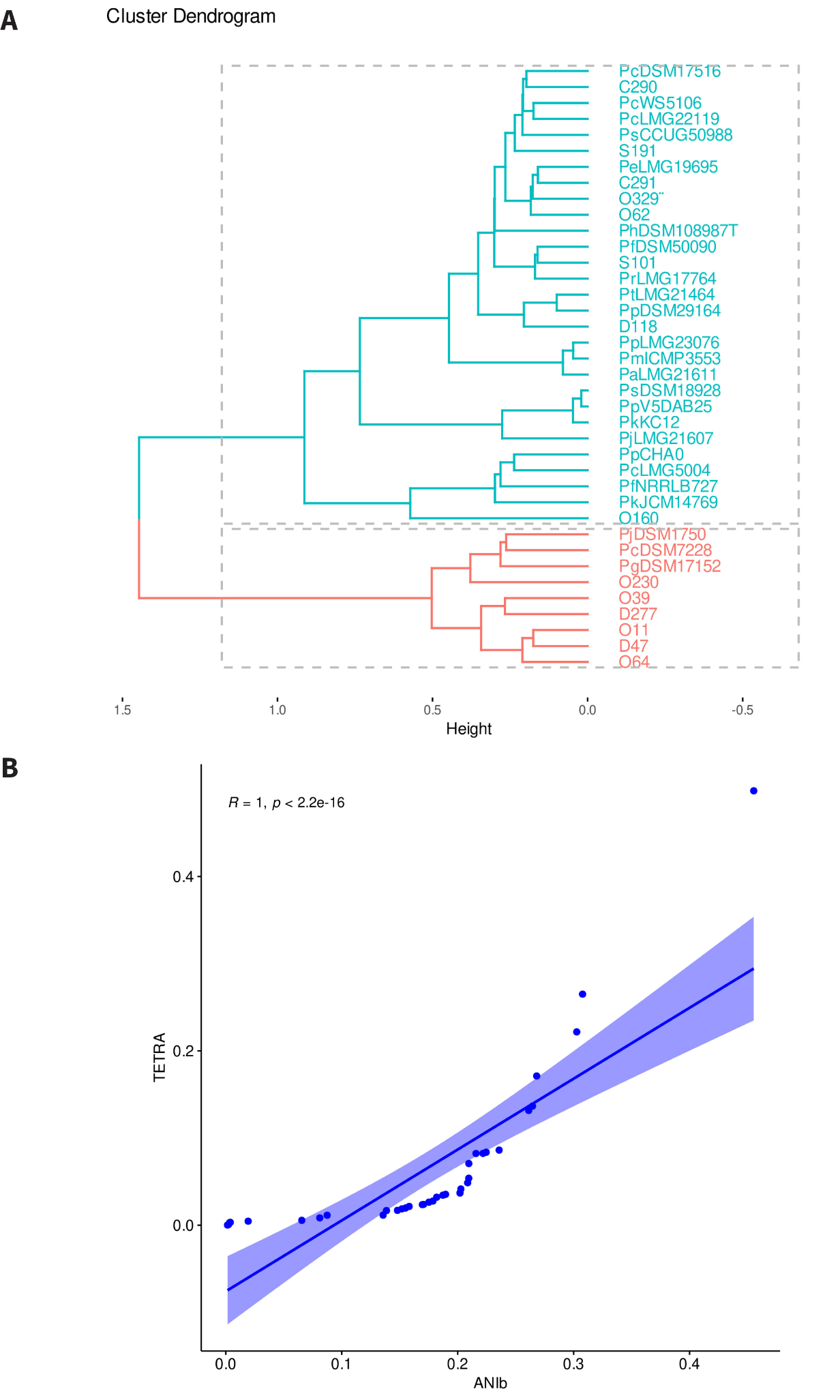


**Supplementary Figure 2: Phylogenomic analysis revealed the formation of two clusters (blue and red). A)** Dendrogram generated using Pyani (Pritchard 2016). The first cluster comprised the highest number of isolates, revealing that C291, O329, and O62 share a recent common ancestor, whereas C290, S191, S101, D118, and O160 were distinct. The second cluster demonstrated that the isolates O230, O39, D277, O11, D47, and O64 had fewer divergences. PcDSM17516: *P. cedrina* type strain DSM 17516; PcWS5106: *P. cremoris* WS 5106; PcLMG22119: *P. costantinii* LMG 22119; PsCCUG50988: *P. simiae* CCUG 50988T; PeLMG19695: *P. extremorientalis* LMG 19695; PhDSM108987T: *P. haemolytica* DSM 108987T; PfDSM50090: *P. fluorescens* DSM 50090; PrLMG17764: *P. rhodesiae* LMG 17764; PtLMG21464: *P. trivialis* LMG 21464; PpDSM29164: *P. paralactis* DSM 29164; PpLMG23076: *P. palleroniana* LMG 23076; PmICMP3553: *P. marginalis* ICMP 3553; PaLMG21611: *P. azotoformans* LMG 21611; PsDSM18928: *P. synxantha* DSM 18928; PpV5DAB25: *P. paracarnis* V5/DAB/2/5; pkKC12: *P. kairouanensis* KC12; PjLMG21607: *P. mandelii* LMG 21607; ppCHA0: *P. protegens* CHA0; PcLMG5004: *P. chlororaphis* LMG 5004; PfNRRLB727: *P. fragi* NRRL B-727; PkJCM14769: *P. koreensis* JCM 14769; PjDSM1750: *P. jessenii* DSM 17150; PcDSM7228: *P. corrugata* DSM 7228; PgDSM17152: *P. gessardii* DSM 17152. **B)** Graphic of the relationship between average nucleotide identity (ANIb) and the correlation indices of tetra-nucleotide (TETRA). Variation occurred between 0.0 and 0.3, with one outlier after 0.4 (*R* = 1, *p* ˂ 2.2e-16).


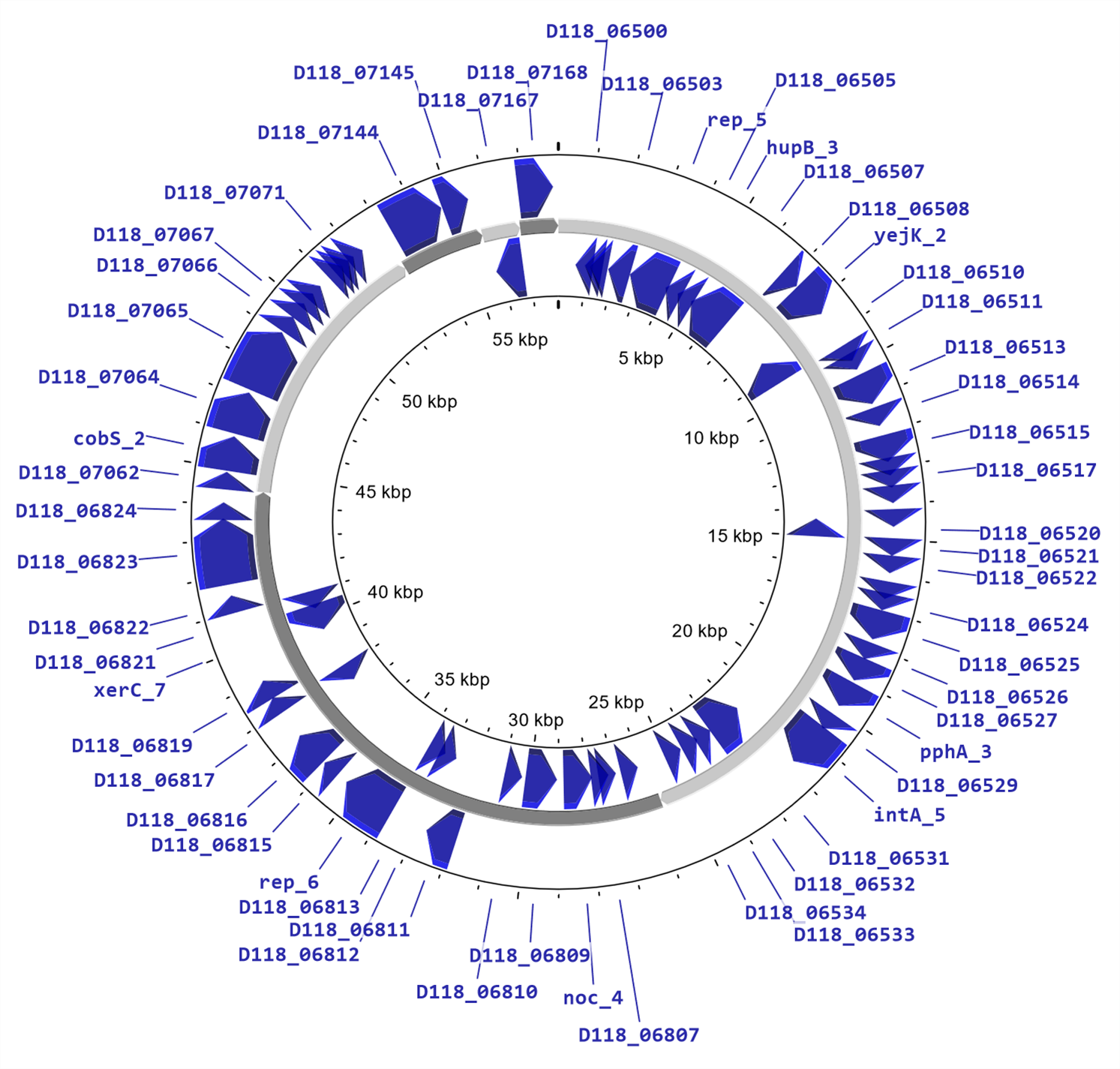


**Supplementary Figure 3: Plasmid identified for isolate D118 (*P. grimontii*).** Open reading frames are represented as blue arrows and do not contain antimicrobial resistance determinants.


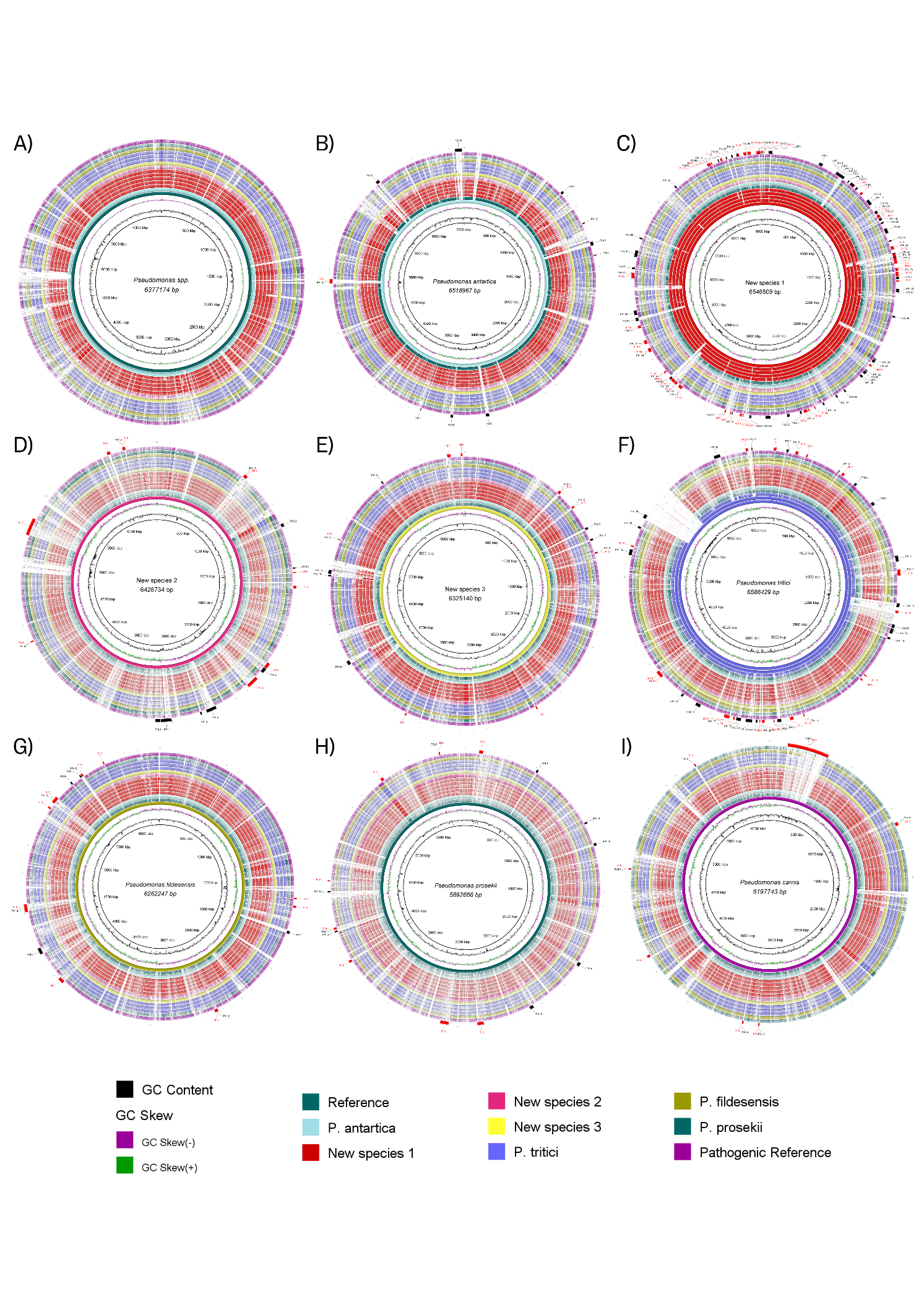
**Supplementary Figure 4:** **Genomic ring plots comparing different *Pseudomonas* species and strains.** Each circular plot uses a distinct genome as the central reference ring to highlight similarities and differences among the strains. Solid colors represent higher similarity (>90%), while faded regions indicate lower similarity (<70%). **A)** Reference for the genus *Pseudomonas*. **B)** Reference strain *P. antarctica* LMG 22709 (NZ_LT629704.1); **C-H)** genomic rings of isolates described in this work; and, **I)** pathogenic reference *P. carnis* BML-PP010 (BQHE01000001.1).

## Supplementary Tables

**Supplementary Table 1:** Phenotypic analysis of 19 antimicrobial agents using the disk diffusion method.

|  | **Disc diffusion (mm), broth microdilution (for CL; µg/mL), and agar dilution (in brackets; µg/mL)** | | | | | | | | | | | | | | | | | | |
| --- | --- | --- | --- | --- | --- | --- | --- | --- | --- | --- | --- | --- | --- | --- | --- | --- | --- | --- | --- |
|  | **AM** | **AMC** | **CF** | **CTX** | **ETP** | **SXT** | **NEO** | **C** | **TE** | **PIP** | **TZP** | **CAZ** | **FEP** | **AZM** | **IPM** | **GM** | **CIP** | **NX** | **CL** |
| **O11*** | 6 | 6 | 6 | 16 | 8 | 6 | 20  (8) | 18 | 28 | 31 | 33 | **14**  **(128)** | **14**  **(128)** | **6**  **(128)** | 25 | 26 | 33 | 32 | **≥256** |
| **O30** | 6 | 6 | 6 | 6 | 11 | 13 | 23  (4) | 10 | 29 | 30 | 30 | **15**  **(128)** | **6**  **(128)** | **6**  **(128)** | 22 | 26 | 30 | 29 | **≥256** |
| **O39*** | 6 | 6 | 6 | 27 | 11 | 21 | 23  (2) | 12 | 30 | 37 | 38 | 30 | 22 | **10**  **(128)** | 20 | 27 | 34 | 34 | **128** |
| **O43** | 6 | 6 | 6 | 16 | 6 | 20 | 23  (4) | 15 | 31 | 36 | 36 | 30 | 24 | **16**  **(128)** | 21 | 27 | 33 | 33 | **128** |
| **D47*** | 6 | 6 | 6 | 14 | 14 | 15 | 21  (4) | 10 | 25 | 32 | 35 | **6**  **(128)** | **6**  **(128)** | **6**  **(128)** | 26 | 27 | 35 | 36 | **≥256** |
| **O58** | 6 | 6 | 6 | 6 | 10 | 13 | 20  (4) | 13 | 28 | 30 | 26 | **6**  **(≥256)** | **6**  **(≥256)** | **6**  **(≥256)** | 25 | 27 | 35 | 32 | **≥256** |
| **O62*** | 6 | 6 | 6 | 6 | 6 | 16 | 23  (4) | 15 | 28 | 30 | 24 | **6**  **(≥256)** | **8**  **(≥256)** | **6**  **(128)** | **16** | 36 | 34 | 32 | **≥256** |
| **O64*** | 6 | 6 | 6 | 6 | 6 | 6 | 26  (4) | 17 | 39 | 36 | 38 | 20 | **16**  **(64)** | **6**  **(128)** | 22 | 30 | 39 | 36 | **≥256** |
| **S101*** | 6 | 6 | 6 | 6 | 14 | 6 | 24  (4) | 13 | 23 | 33 | 34 | **6**  **(≥256)** | 23 | **6**  **(128)** | 30 | 27 | 37 | 36 | **≥256** |
| **D118*** | 6 | 6 | 6 | 6 | 20 | 9 | 22  (4) | 11 | 25 | 31 | 30 | 22 | 20 | **6**  **(≥256)** | 35 | 28 | 40 | 35 | **2** |
| **O157** | 6 | 11 | 6 | 14 | 30 | 6 | 25  (4) | 15 | 27 | 28 | 28 | **12**  **(64)** | 24 | **6**  **(128)** | 29 | 25 | 37 | 34 | **0.5** |
| **O160*** | 6 | 12 | 6 | 11 | 23 | 6 | 22  (2) | 6 | 25 | 30 | 30 | **12**  **(64)** | 26 | **6**  **(128)** | 34 | 24 | 39 | 36 | **0.5** |
| **C181** | 6 | 12 | 6 | 13 | 29 | 6 | 21  (2) | 13 | 27 | 34 | 34 | **13**  **(64)** | 30 | **12**  **(128)** | 32 | 29 | 40 | 38 | **1** |
| **C187** | 6 | 11 | 6 | 14 | 29 | 6 | 24  (2) | 12 | 29 | 28 | 30 | **12**  **(≥256)** | 26 | **8**  **(128)** | 30 | 27 | 36 | 36 | **0.5** |
| **S191*** | 6 | 6 | 6 | 6 | 12 | 6 | 21  (4) | 16 | 27 | 31 | 32 | **17**  **(≥256)** | 18 | **6**  **(128)** | 27 | 27 | 38 | 32 | **≥256** |
| **O230*** | 6 | 6 | 6 | 24 | 37 | 6 | 21  (4) | 18 | 32 | 30 | 32 | 27 | 28 | **6**  **(128)** | 38 | 22 | 40 | 38 | **1** |
| **C252** | 6 | 6 | 6 | 19 | 16 | 14 | 19  (8) | 14 | 21 | 26 | 24 | 20 | 20 | **6**  **(256)** | 19 | 14 | 29 | 27 | **≥256** |
| **D273** | 6 | 6 | 6 | 10 | 19 | 6 | 21  (8) | 6 | 26 | 30 | 30 | 27 | 24 | **6**  **(128)** | 30 | 28 | 40 | 37 | **1** |
| **D277*** | 6 | 6 | 6 | 11 | 13 | 6 | 22  (8) | 10 | 28 | 30 | 29 | 23 | 23 | **6**  **(128)** | 25 | 27 | 37 | 34 | **2** |
| **C290*** | 6 | 6 | 6 | 9 | 13 | 6 | 20  (4) | 16 | 21 | 29 | 30 | 22 | 26 | **6**  **(128)** | 30 | 25 | 40 | 36 | **4** |
| **C291*** | 6 | 6 | 6 | 9 | 16 | 11 | 22  (8) | 9 | 22 | 32 | 39 | 22  (32) | 22 | **6**  **(128)** | 30 | 25 | 40 | 34 | **1** |
| **C294** | 6 | 6 | 6 | 10 | 16 | 6 | 22  (8) | 12 | 28 | 33 | 30 | 24 | 24 | **6**  **(128)** | 28 | 24 | 37 | 30 | **1** |
| **O329*** | 6 | 6 | 6 | 6 | 12 | 6 | 20  (4) | 6 | 30 | 31 | 31 | 25 | 25 | **6**  **(128)** | 32 | 23 | 33 | 32 | **1** |
| **O331** | 6 | 6 | 6 | 11 | 13 | 6 | 19  (4) | 12 | 26 | 28 | 28 | 24 | 24 | **6**  **(128)** | 30 | 20 | 31 | 29 | **1** |
| **O332** | 6 | 6 | 6 | 11 | 12 | 6 | 20  (4) | 12 | 31 | 35 | 38 | 28 | 28 | **6**  **(128)** | 35 | 24 | 37 | 36 | **0.5** |
| ***P. aeruginosa***  **ATCC 27853** | 6 | 6 | 6 | 24 | 25 | 6 | 6  (≥8) | 6 | 23 | 33 | 32 | 30  (4) | 30  (8) | 30  (4) | 34 | 25 | 28 | 24 | 2 |
| ***E. coli***  **ATCC 25922** | n.d. | n.d. | n.d. | n.d. | n.d. | n.d. | n.d.  (4) | n.d. | n.d. | n.d. | n.d. | n.d. | n.d. | n.d. | n.d. | n.d. | n.d. | n.d. | 1 |
| ***E. coli* C153** | n.d. | n.d. | n.d. | n.d. | n.d. | n.d. | n.d. | n.d. | n.d. | n.d. | n.d. | n.d. | n.d. | n.d. | n.d. | n.d. | n.d. | n.d. | 8 |

The relevant intermediate/resistant phenotypes against antimicrobials available for the treatment of *Pseudomonas* are indicated in bold. When the results were discrepant in comparison to the positive control *P. aeruginosa* ATCC 27853 and CLSI standards (CLSI 2023), MIC using agar diffusion was also performed. The MIC using broth microdilution was performed for colistin. * isolates selected for whole genome sequencing. AM: ampicillin; AMC: amoxicillin-clavulanate; CF: cephalothin; CTX: cefotaxime; ETP: ertapenem; SXT: sulfamethoxazole-trimethoprim; NEO: neomycin; C: chloramphenicol; TE: tetracycline; PIP: piperacillin; TZP: piperacillin-tazobactam; CAZ: ceftazidime; FEP: cefepime; AZM: aztreonam; IPM: imipenem; GM: gentamicin; CIP: ciprofloxacin; NX: norfloxacin; CL: colistin; n.d.: non-defined.

**Supplementary Table 2:** Nucleotide accession number of genomes from the *Pseudomonas fluorescens* group for phylogenomic analysis.

| **Species** | **Nucleotide accession number** |
| --- | --- |
| *P. cedrina* type strain DSM 17516 | NZ_MNPW01000010.1 |
| *P. cremoris* WS 5106 | NZ_JAAXCY010000010.1 |
| *P. costantinii* LMG 22119 | NZ_FNTS01000002.1 |
| *P. simiae* CCUG 50988T | NZ_MDFH01000001.1 |
| *P. extremorientalis* LMG 19695 | NZ_MDGK01000001.1 |
| *P. haemolytica* DSM 108987T | NZ_VOIW01000010.1 |
| *P. fluorescens* DSM 50090 | LHVP01000001.1 |
| *P. rhodesiae* LMG 17764 | NZ_LT629801.1 |
| *P. trivialis* LMG 21464 | NZ_LT629760.1 |
| *P. paralactis* DSM 29164 | NZ_JYLN01000010.1 |
| *P. palleroniana* LMG 23076 | NZ_FNUA01000002.1 |
| *P. marginalis* ICMP 3553 | NZ_LKEG01000001.1 |
| *P. azotoformans* LMG 21611 | NZ_LT629702.1 |
| *P. synxantha* DSM 18928 | NZ_JYLJ01000010.1 |
| *P. paracarnis* V5/DAB/2/5 | NZ_CAJFCN010000001.1 |
| *P. kairouanensis* KC12 | NZ_QUZU01000001.1 |
| *P. mandelii* LMG 21607 | LT629796.1 |
| *P. protegens* CHA0 | NZ_LS999205.1 |
| *P. chlororaphis* LMG 5004 | LHVC01000001.1 |
| *P. fragi* NRRL B-727 | NZ_LT629783.1 |
| *P. koreensis* JCM 14769 | BMOG01000001.1 |
| *P. jessenii* DSM 17150 | NIWT01000001.1 |
| *P. corrugata* DSM 7228 | LHVK01000001.1 |
| *P. gessardii* DSM 17152 | NZ_MNPU01000100.1 |
| *P. grimontii* DSM 17515 | GCA_900101085.1 |
| *P. marginalis* ICMP 3553 | NZ_LKEG01000001.1 |
| *P. tritici* SWRI 145 | GCA_014268275.3 |
| *P. antarctica* LMG 22709 | GCA_900103795.1 |
| *P. fildesensis* KG01 | GCA_001050345.1 |
| *P. prosekii* LMG 26867 | GCA_900105155.1 |

**Supplementary Table 3:** Annotation of antimicrobial resistance genes using the ABRicate v.1.0.1 pipeline with NCBI AMRFinderPlus v.3.12.8 (Feldgarden et al. 2021; Feldgarden et al. 2022), MEGARes v.3.0 (Lakin et al. 2017; Bonin et al. 2023), and CARD v.3.2.4 (Alcock et al. 2023; McArthur et al. 2013). The table displays the raw data from the database searches, including percent coverage, identity, coordinates, and additional annotations.

**Supplementary Table 4:** Taxonomic description of the fourteen isolates described in this work. All of the isolates belonged to the *P. fluorescens* complex. Furthermore, three potential new species were identified.

**References**

Alcock, B. P., W. Huynh, R. Chalil, K. W. Smith, A. R. Raphenya, M. A. Wlodarski, A. Edalatmand, A. Petkau, S. A. Syed, K. K. Tsang, S. J. C. Baker, M. Dave, M. C. McCarthy, K. M. Mukiri, J. A. Nasir, B. Golbon, H. Imtiaz, X. Jiang, K. Kaur, M. Kwong, Z. C. Liang, K. C. Niu, P. Shan, J. Y. J. Yang, K. L. Gray, G. R. Hoad, B. Jia, T. Bhando, L. A. Carfrae, M. A. Farha, S. French, R. Gordzevich, K. Rachwalski, M. M. Tu, E. Bordeleau, D. Dooley, E. Griffiths, H. L. Zubyk, E. D. Brown, F. Maguire, R. G. Beiko, W. W. L. Hsiao, F. S. L. Brinkman, G. Van Domselaar, and A. G. McArthur. 2023. 'CARD 2023: expanded curation, support for machine learning, and resistome prediction at the Comprehensive Antibiotic Resistance Database', *Nucleic Acids Res*, 51: D690-D99.

Bonin, N., E. Doster, H. Worley, L. J. Pinnell, J. E. Bravo, P. Ferm, S. Marini, M. Prosperi, N. Noyes, P. S. Morley, and C. Boucher. 2023. 'MEGARes and AMR++, v3.0: an updated comprehensive database of antimicrobial resistance determinants and an improved software pipeline for classification using high-throughput sequencing', *Nucleic Acids Res*, 51: D744-D52.

CLSI, Clinical Laboratory Standards Institute 2023. *Performance Standards for Antimicrobial Susceptibility Testing, M100,* .

Feldgarden, M., V. Brover, B. Fedorov, D. H. Haft, A. B. Prasad, and W. Klimke. 2022. 'Curation of the AMRFinderPlus databases: applications, functionality and impact', *Microb Genom*, 8.

Feldgarden, M., V. Brover, N. Gonzalez-Escalona, J. G. Frye, J. Haendiges, D. H. Haft, M. Hoffmann, J. B. Pettengill, A. B. Prasad, G. E. Tillman, G. H. Tyson, and W. Klimke. 2021. 'AMRFinderPlus and the Reference Gene Catalog facilitate examination of the genomic links among antimicrobial resistance, stress response, and virulence', *Sci Rep*, 11: 12728.

Lakin, S. M., C. Dean, N. R. Noyes, A. Dettenwanger, A. S. Ross, E. Doster, P. Rovira, Z. Abdo, K. L. Jones, J. Ruiz, K. E. Belk, P. S. Morley, and C. Boucher. 2017. 'MEGARes: an antimicrobial resistance database for high throughput sequencing', *Nucleic Acids Res*, 45: D574-D80.

McArthur, A. G., N. Waglechner, F. Nizam, A. Yan, M. A. Azad, A. J. Baylay, K. Bhullar, M. J. Canova, G. De Pascale, L. Ejim, L. Kalan, A. M. King, K. Koteva, M. Morar, M. R. Mulvey, J. S. O'Brien, A. C. Pawlowski, L. J. Piddock, P. Spanogiannopoulos, A. D. Sutherland, I. Tang, P. L. Taylor, M. Thaker, W. Wang, M. Yan, T. Yu, and G. D. Wright. 2013. 'The comprehensive antibiotic resistance database', *Antimicrob Agents Chemother*, 57: 3348-57.

Pritchard, L.; Glover, R.H.; Humphris, S.; Elphinstone, J.G.; Toth, I.K. 2016. 'Genomics and taxonomy in diagnostics for food security: soft-rotting enterobacterial plant pathogens', *Anal. Methods*, 8: 12-24.
